# A case of GFAP-IgG positivity followed by anti-NMDAR encephalitis

**DOI:** 10.1186/s12887-022-03650-2

**Published:** 2022-10-17

**Authors:** Jia Zhang, Jing Gan, Jianjun Wang

**Affiliations:** 1 Present Address: Department of Pediatrics, West China Second University Hospital, Sichuan University, No. 20, Section Three, South Renmin Road, Chengdu, 610041 China; 2 Key Laboratory of Obstetrics & Gynecologic and Pediatric Diseases and Birth Defects of the Ministry of Education, Sichuan University, Chengdu, Sichuan China; 3 Key Laboratory of Development and Maternal and Child Diseases of Sichuan Province, Chengdu, Sichuan China

**Keywords:** Anti-NMDAR encephalitis, GFAP, Autoimmune encephalitis, Antibody overlapping syndrome

## Abstract

**Background:**

In recent years, there have been an increasing number of reports on overlapping antibodies in autoimmune encephalitis (AE). There are various types of overlapping antibodies, but the clinical significance of each type is not yet clear. Glial antibodies, such as MOG, AQP4, and especially NMDAR, can be detected in patients with AE. However, little is known about the overlapping antibodies of anti-glial fibrillary acidic protein (GFAP), and only a few case reports have described this overlap.

Case presentation

The patient was a 7-year-old girl with recurrent intermittent fever and seizures, and viral encephalitis was diagnosed at the beginning of the disease. She was discharged after treatment with acyclovir, high-dose immunoglobulins, and valproic acid as an antiseizure medication. Subsequently, the patient still had occasional seizures and abnormal behavior, and the anti-NMDAR antibody test was positive (1:3.2). She was treated with high-dose methylprednisolone and antiseizure therapy. Approximately half a year later, the patient experienced fever and seizures again, serum GFAP IgG was 1:100, and a head MRI indicated new lesions. Improvement was achieved after repeated high-dose methylprednisolone and continuous prednisone anti-inflammatory therapy.

**Conclusions:**

Anti-NMDAR encephalitis combined with GFAP-IgG is uncommon, and repeated tests for AE-associated antibodies may be required in patients with recurrent encephalitis. Compared with cerebrospinal fluid antibody-positive children, serum GFAP IgG-positive children should be comprehensively diagnosed according to their clinical manifestations. It is worth considering whether overlapping antibody syndrome can still be an issue for patients with AE who recover and have negative antibodies after a few months if disease recurrence and new antibodies are detected.

## Background

Autoimmune encephalitis (AE) is an autoimmune disease of the central nervous system that is mediated by anti-neuron antibodies or antibody-related molecules. Many antibody-associated neuroimmune syndromes have been recognized as diseases with the expansion of antibody lineages and the discovery of new autoimmune antibodies in the understanding of AE. At the same time, the fact that some AE cases have multiple positive antibodies or phenotype overlap has aroused clinical attention, but the mechanism underlying these observations needs to be further studied. The clinical data of a child with anti-NMDAR encephalitis relapse are reported as follows.

## Case presentation

**Fig. 1:**
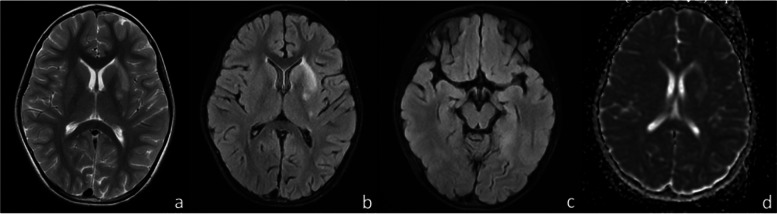
Head MRI (**a** T2WI axial image, **b**, **c** T2-FLAIR axial image, **d** DWI axial image) showed a slightly longer patchy T1 and slightly longer T2 abnormal signal in the left basal ganglia, T2-FLAIR image showed hyperintensity, and DWI image showed no diffuse limited hyperintensity in the skull. Hyperintensity patches on the left cauda equina, bilateral frontotemporal cortex, cingulate gyrus were observed on local T2-Flair with unclear boundaries, but no hyperintensity patches were observed on DWI. No enhancement was observed in the enhancement scan

The child is a 7 + year-old girl. On August 30, 2020, she presented with fever and a seizure, which was categorized as a GTCS (generalized tonic‒clonic seizure) lasting approximately 1–2 min. At the beginning of the disease, there was vomiting, dizziness, headache, and other symptoms. Cerebrospinal fluid (CSF) analysis done on September 2 showed that the WBC were 30 × 10^6/L, of which mononuclear cells were 53%, polynuclear cells were 47%. CSF biochemistry, smear, and culture showed no abnormalities. Head MRI showed abnormal signals in the right caudate nucleus and lenticular nucleus. After 2 weeks of anti-infection treatment with cefotaxime sodium and ceftriaxone successively and antiseizure treatment with valproic acid in another hospital, the patient was discharged. After discharge, the patient had another seizure of the same type as before. She was admitted to our hospital on September 14, 2020, during which she had one more seizure. Physical examination of the nervous system and other organ systems showed no abnormalities. There was no special past or family history. CSF analysis on September 15 showed a nucleated cell count of 20 × 10^6/L, neutral lobulation was 42%, lymphocytes were 50%, and monocytes were 8%. CSF biochemistry, smear, and culture showed no abnormalities. AE-associated antibodies and metagenomic next-generation sequencing (mNGS) were normal. Cranial vascular enhanced MRI on September 17 showed abnormal signals in the left basal ganglia, hippocampus, cingulate gyrus, and bilateral frontotemporal cortex, suggesting inflammatory changes. No obvious abnormality was observed in MRA and MRV (Fig. 1). Video electroencephalogram (VEEG) on September 21 showed that background waves were decreased (5–7 Hz), and multifocal δ slow waves were emitted multiple times. She was treated with Ceftriaxone for the infection, acyclovir as an antiviral agent, mannitol to reduce cranial pressure, large-dose immunoglobulin (1 g/kg × 2 d), dexamethasone to decrease inflammation, valproic acid to treat seizures, and vitamin B for 10 days, and then discharged.

After discharge, the child still had occasional seizures and abnormal behavior manifested as speaking gibberish, repetitive speech, and exhalation, which lasted 1–2 h. She still had an intermittent fever, headache, and vomiting and was readmitted to our hospital on January 1, 2021. The nucleated cell count of CSF was 10 × 10^6/L, neutral lobules were 83%, lymphocytes were 16%, monocytes were 1% and CSF biochemistry, smear, and culture showed no abnormalities. Serum and CSF AE-associated antibodies were negative. Autoantibodies, tumor markers, and cellular/humoral immunity were normal. VEEG on January 6 showed that background waves were slow (4–7 Hz), and multifocal δ slow waves were frequently discharged. The visual evoked potentials showed abnormalities (left eye, right eye, and both eyes were stimulated, the latency of the P100 wave was prolonged, and the amplitude of the wave was normal). Head MRI showed that the posterior segment of the bilateral optic nerve was slightly thickened, the signal on T2WI was slightly increased, and the enhancement was fairly uniform. No clear abnormalities were observed in the rest of the brain parenchyma. The count of nucleated cells in the CSF on January 15 was 33 × 10^6/L, 64% were neutral lobules, 34% were lymphocytes, 2% were monocytes, and 2.42 mmol/L sugar. No abnormality was found in the smear or culture. Anti-NMDAR antibody IgG in blood and cerebrospinal fluid was positive (1:3.2). Chest and abdominal CT showed no abnormalities. Large-dose immunoglobulin (1 g/kg × 2 d) and methylprednisolone (20 mg/kg × 3 d), phenobarbital, and valproic acid treatment improved after discharge. After discharge, prednisone and valproic acid (25 mg/kg.d) were administered regularly, and no symptoms were observed. Reexamination of NMDAR IgG was negative more than 3 months later. Re-examination of VEEG showed no abnormalities.

**Fig. 2:**
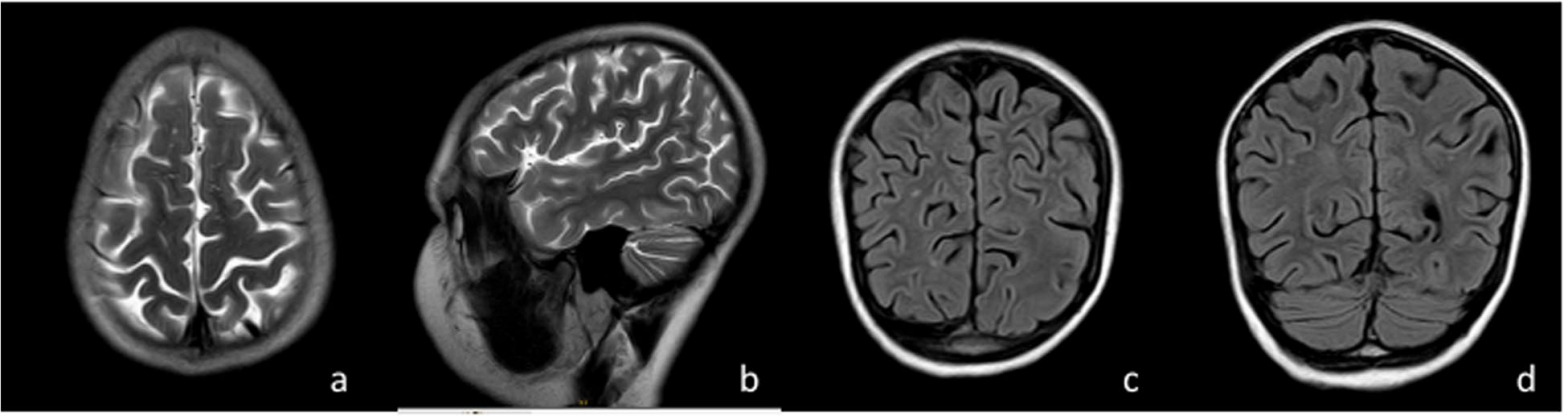
Head MRI (**a** T2WI axial image, **b** T2WI sagittal image, **c** T2-FLAIR axial image, **d** T2-FLAIR coronal image) showed multiple spot-like and nodular abnormal signals in both cerebral hemispheres, slightly widened bilateral lateral ventricles, and slightly deepened sulci in both cerebral and cerebellar hemispheres

**Fig. 3:**
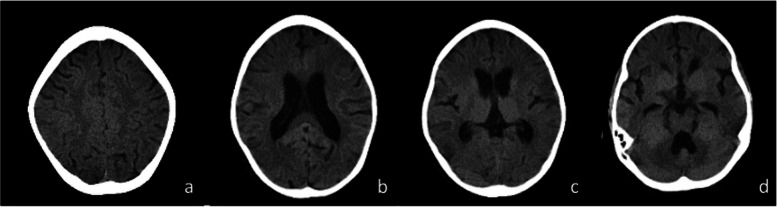
Head CT showed slightly lower parenchyma density in both cerebral hemispheres and an unclear intracranial gray matter boundary. Sulci slightly widened and deepened, bilateral lateral ventricles widened, posterior fossa pool widened

On September 27, 2021, the follow-up examination of head MRI showed multiple spotted and nodular abnormal signal shadows in both cerebral hemispheres, slightly widened bilateral lateral ventricles and slightly deepened sulci in both cerebral hemispheres and cerebellar hemispheres. Compared with the scan from January 06, these changes were new (Fig. 2). On October 9th, the child had fever and another seizure, which manifested as twitching of the right corner of the mouth, accompanied by involuntary shaking of the right upper limb, and loss of consciousness, which lasted for approximately 1 min. No abnormalities were found in serum and CSF inflammatory indices or pathogen screening. Serum GFAP IgG was positive (1:100), and ANA was 1:320. No abnormalities were observed in thyroid function or antibodies. An EEG on October 13 suggested a background wave of 9–10 Hz, and slow waves were frequently emitted from the left middle and posterior temporal regions. Head CT on October 16 showed that the density of the parenchyma in the bilateral cerebral hemispheres was slightly lower, and the intracranial gray matter boundary was not clear. The sulci were slightly widened and deepened, the bilateral lateral ventricles widened, and the posterior fossa pool widened (Fig. 3). Visual evoked potentials on October 19 showed that the latency of the P100 wave was prolonged in both eyes. The auditory evoked potential of 120 d BPeSPL showed prolonged latency of left waves and prolonged latency of right IV and V waves. The patient was discharged after treatment with large-dose methylprednisolone and continuous prednisolone anti-inflammatory therapy. Serum and CSF GFAP IgG were negative after 1 month. Three months later, head MRI showed no obvious abnormalities, seizures, disturbances of consciousness, or other changes. At present, the patient has been followed up for approximately six months, the dosage of prednisone acetate was reduced to 5 mg daily, and valproic acid taken orally at 5 mg/kg daily, without seizures or disturbances of consciousness.

## Discussion and conclusions

The discovery of anti-N-methyl-D-aspartate receptor (NMDAR) encephalitis in 2007 opened a new chapter of clinical research on AE. At present, AE is classified anatomically, serologically, and etiologically [1], but the clinical serological classification is the main classification. With the expansion of anti-neuronal antibody lineages and the improvement of antibody detection methods, the phenomenon of multiple anti- neuronal antibody positivity in AE has attracted attention. However, its clinical significance remains unclear. Titulaer et al. reported that in 691 cases of anti-NMDAR encephalitis, AQP4, or MOG IgG were detected in serum or CSF in 23 (4%) [2]. In the 220 anti-NMDAR encephalitis cohort reported by Xu et al., 11 cases (5%) were combined with glial cell antibodies, including 5 MOG cases and 4 AQP4 cases [3]. GFAP is a cytoplasmic antigen associated with intermediate filaments. Currently, there are relatively few reports on overlapping GFAP with anti-NMDAR encephalitis, and most patients have atypical symptoms. The diagnostic significance of overlapping antibodies is unclear. Clinical phenotype and antibodies are the two elements of the diagnosis of AE, neither of which is indispensable, and an antibody-positive diagnosis of AE cannot be relied on alone.

Martinez-Hernandez et al. screened the CSF and serum of 846 patients with anti-NMDAR encephalitis and found that 10 patients were simultaneously positive for anti-GFAP antibody [4]. In a study of 102 patients with GFAP, Flanagan et al. found that approximately 22 patients (22%) also had anti-NMDAR antibodies [5]. In addition to the typical symptoms of anti-NMDAR encephalitis, these patients either had or did not have symptoms related to glial fibrillary acid protein astrocytoma (GFAP-A), and most of them did not have the typical imaging characteristics of GFAP-A. The clinical manifestations of GFAP-A are not specific, and the diagnostic criteria are not uniform. At present, the overlapping syndrome of AE is mainly based on the presence of overlapping antibodies. As reported thus far, overlapping NMDARs with GFAP is not uncommon. Both GFAP and NMDAR, and possibly AQP4, are expressed in teratoma, with the highest predictive value (71%) when GFAP is combined with NMDAR and AQP4. Plasma membrane protein-directed IgG in AQP4 and NMDAR may trigger primary inflammatory events and disrupt astrocyte function, and GFAP IgG may occur as a secondary phenomenon [4]. Therefore, the international guidelines recommend that all children with anti-NMDAR encephalitis undergo tumor searches for ovarian teratoma and other tumors, which should be initiated as early as possible and completed within the first few days to weeks after admission to hospital [6]. In this study, chest, and abdominal CT examinations were completed when NMDAR antibody positivity was found.

GFAP is a biomarker of astrocytes and is involved in many biological functions of astrocytes. GFAP-A is an autoimmune-mediated new central nervous system disease mainly involving the meninges, brain, spinal cord, and optic nerve, with fever, headache, encephalopathy, myelitis, and optic neuritis as the main clinical features. The disease was first reported by the Mayo Clinic in 2016, and GFAP antibodies were identified as biomarkers for the disease [7]. Due to the different distribution of antibodies, the distribution of different types of overlapping antibodies in serum and CSF is also different. GFAP IgG is a cytoplasmic antibody that, unlike AQP4, binds to cell surface antigens to play a direct pathogenic role and is only a biomarker of the inflammatory process of CD8 + T cells [8]. GFAP has at least eight isomers. Therefore, it is difficult to detect all isomers by the CBA method, and α and ε isomers are mainly detected at present. Most studies show that the sensitivity and specificity of GFAP IgG in CSF are higher than those in serum. The diagnosis of GFAP-A should be based on the clinical symptoms, imaging, EEG characteristics, and treatment response. GFAP antibody is positive in the serum of some patients before positive detection in CSF, which may be due to the occurrence of peripheral nerve injury earlier than injury in the central nervous system [9]. In this case, the patient started with fever, headache, and seizures. At the beginning of the disease, multiple tests of AE antibodies were negative. Later, psychiatric and behavioral abnormalities appeared, and NMDAR antibody was positive again. However, GFAP antibody was not reexamined at that time. It was thought that the child had optic nerve inflammation, and only GFAP IgG was positive in subsequent relapses of the disease. We cannot rule out the possibility that anti-NMDAR antibodies were combined with GFAP IgG at that time. Optic neuritis/abnormal vision is not common in patients with anti-NMDAR encephalitis in the clinic. Although only serum GFAP IgG was positive in this case, there was a new intracranial lesion, CSF inflammatory changes, and immunotherapy was effective. Therefore, it is suggested that the possibility of overlapping antibodies should be considered when patients with AE have clinical phenotypes that cannot be routinely explained, and the antibody detection spectrum should be appropriately amplified according to the clinical phenotypes. Approximately 50% of patients with overlapping antibodies may have subsequent AE recurrence, suggesting the persistence of immune abnormalities in patients [3, 4]. In addition, in the early stage of AE, head MRI and antibodies may be negative, and antibodies can be detected under certain infections and other inducing factors [1]. Hence, repeated detection of AE antibodies may be required in some patients. Intracranial infection should be excluded in the diagnosis of AE, and other systemic autoimmune diseases should be considered when the antibody test is negative. In this study, infection was investigated at the beginning, and the subsequent condition was repeated. The AE antibody test was negative, and further screening for thyroid antibodies and autoantibodies and other examinations were performed.

At present, the diagnosis of overlapping antibody syndrome involves detecting multiple antibodies at the same time. Among the 35 children with GFAP-A reported by Fang et al., 11 (31.4%) were positive for other antibodies, and one patient developed autoimmune GFAP-A 1 year after the diagnosis of AQP4 antibody-associated neuromyelitis optica (NMO). One MOGAD patient was found to be positive for antibodies to GFAP several months after treatment [9]. Of the 30 GFAP IgG-positive patients reported by Yang et al., 10 had overlap syndrome, with one patient developing GFAP-A 10 years after diagnosis of NMO and the other diagnosed GFAP-A 1 year after diagnosis of anti-NMDAR encephalitis [10], similar to this patient. Therefore, it is worth considering whether overlapping antibody syndrome can still be the underlying cause for patients with AE whose condition improves and the negative antibody is reexamined several months later if the subsequent condition repeats and new antibodies are detected. At present, Yamakawa et al. reported a patient whose clinical diagnosis was fully consistent with GFAP-A. Including the head MRI, which showed linear perivascular enhancement around the ventricle, the autopsy results showed nonspecific inflammatory changes and did not show astrocyte involvement [11]. The anti-GFAP antibody is more like a bystander that witnesses an upstream inflammatory response than a pathogenic antibody that causes a subsequent inflammatory or immune response. Therefore, the clinical causality of anti-GFAP antibodies and their status as diagnostic markers requires further clinical evaluation, including clinicopathological evaluation.

In summary, this study has some limitations. It is a case report of retrospective analysis, and GFAP was not detected during the first reexamination of the NMDAR antibody. However, it provides new directions for the clinic. First, for patients with recurrent encephalitis, AE-associated antibodies may be tested several times, and the AE-associated antibody spectrum should be appropriately increased according to the clinical phenotype of patients to improve the diagnosis and treatment of their disease. Second, due to different antibody distributions, compared with CSF antibody positivity, children with serum GFAP IgG positivity should be comprehensively assessed according to their clinical manifestations. Both clinical phenotype and antibody levels are indispensable. Finally, it is worth considering whether overlapping antibody syndrome can considered for patients with AE who have recovered and whose antibody was negative after reexamination a few months later.

## Data Availability

The original contributions presented in the study are included in the article/supplementary material, further inquiries can be directed to the corresponding author.

## Abbreviations

AE: Autoimmune encephalitis
GFAP: Anti-glial fibrillary acidic protein
GTCS: Generalized tonic–clonic seizure
CSF: Cerebrospinal fluid
mNGS: Metagenomic next-generation sequencing
VEEG: Video electroencephalogram
NMDAR: anti-N-methyl-D-Aspartate receptor
GFAP-A: Glial fibrillary acid protein astrocytoma
